# Measurement of Carcinoembryonic Antigen in Clinical Serum Samples Using a Centrifugal Microfluidic Device

**DOI:** 10.3390/mi9090470

**Published:** 2018-09-17

**Authors:** Zhigang Gao, Zongzheng Chen, Jiu Deng, Xiaorui Li, Yueyang Qu, Lingling Xu, Yong Luo, Yao Lu, Tingjiao Liu, Weijie Zhao, Bingcheng Lin

**Affiliations:** 1 School of Pharmaceutical Science and Technology, Dalian University of Technology, Dalian 116024, China; gzg1980@dlut.edu.cn (Z.G.); dengjiu@mail.dlut.edu.cn (J.D.); xrli@mail.dlut.edu.cn (X.L.); yyqu@mail.dlut.edu.cn (Y.Q.); nongxuexueshi@gmail.com (L.X.); bclin@dicp.ac.cn (B.L.); 2 Integrated Chinese and Western Medicine Postdoctoral research station, Jinan University, Guangzhou 510632, China; chenmond@foxmail.com; 3 Dalian Institute of Chemical Physics, Chinese Academy of Sciences, Dalian 116023, China; luyao@dicp.ac.cn; 4 College of Stomatology, Dalian Medical University, Dalian 116024, China; tingjiao@dlmedu.edu.cn

**Keywords:** centrifugal microfluidic device, CEA detection, density medium, fluorescent chemiluminescence

## Abstract

Carcinoembryonic antigen (CEA) is a broad-spectrum tumor marker used in clinical applications. The primarily clinical method for measuring CEA is based on chemiluminescence in serum during enzyme-linked immunosorbent assays (ELISA) in 96-well plates. However, this multi-step process requires large and expensive instruments, and takes a long time. In this study, a high-throughput centrifugal microfluidic device was developed for detecting CEA in serum without the need for cumbersome washing steps normally used in immunoreactions. This centrifugal microdevice contains 14 identical pencil-like units, and the CEA molecules are separated from the bulk serum for subsequent immunofluorescence detection using density gradient centrifugation in each unit simultaneously. To determine the optimal conditions for CEA detection in serum, the effects of the density of the medium, rotation speed, and spin duration were investigated. The measured values from 34 clinical serum samples using this high-throughput centrifugal microfluidic device showed good agreement with the known values (average relative error = 9.22%). These results indicate that the high-throughput centrifugal microfluidic device could provide an alternative approach for replacing the classical method for CEA detection in clinical serum samples.

## 1. Introduction

Carcinoembryonic antigen (CEA) is a polysaccharide-protein complex with a molecular weight that ranges from 180 to 220 kD, and has 28 potential N-linked glycosylation sites. CEA is primarily produced by the embryonic intestinal mucous membranes prior to birth. Thus, the concentration of CEA is usually very low in the serum of a healthy adult. However, the serum concentration of CEA can become elevated in the presence of several types of cancer, such as lung [1,2], breast [3,4], colorectal [5,6], or gastric [7] cancers, as well as colon adenocarcinoma [8]. This means that CEA can be considered a broad-spectrum biomarker for cancer diagnosis and prognosis.

A variety of immunoassay methods have been developed for detecting CEA in serum, such as enzyme-linked immunosorbent assays (ELISA) [9], radioimmunoassays [10], fluorescence immunoassays [11], chemiluminescence immunoassays [12] and amperometric immunoassays [13]. However, a common drawback of these testing methods is that multiple washing steps are required. These repeated washing steps can give rise to increasing measuring errors, which decreases the efficiency while requiring complex instrumentation. Recently, a wash-free one-step immunoassay [14] was developed using a centrifugal microfluidic device, which has great potential for use in clinical applications. This immunoassay method is based on the principle of centrifugal density gradient equilibrium, which takes place inside a microfluidic device. Analytes with fluorescent labels were separated from the bulk serum in one step, using the centrifugal force, through the dense medium located in the microchannels. Afterwards, the fluorescence microbeads, which aggregated at the end of microchannel, could be collected for quantitative analysis.

Following this strategy, Interleukin 6 was rapidly measured (within 15 min) in whole blood by Ulrich et al. [14]. Chung-Yan Koh et al. accomplished the ultrasensitive detection of botulinum toxin in a 2 μL unprocessed sample in 30 min from sample to answer [15]. These studies showed that it is possible to develop a rapid, accurate, high-throughput centrifugal microfluidic chip for the detection of CEA in serum.

In this study, chitosan, which is safe and has good biocompatibility, was used as the dense medium in the centrifugal microfluidic. When combined with ELISA testing, the CEA could be separated by the action of the centrifugal force produced by the rotation, and the concentration of CEA could be detected using a semi-quantitative fluorescence method. This enables the rapid and convenient detection of CEA in serum with high throughput.

## 2. Materials and Methods

### 2.1. Design and Manufacture of the Centrifugal Microfluidic Device

**Figure 1:**
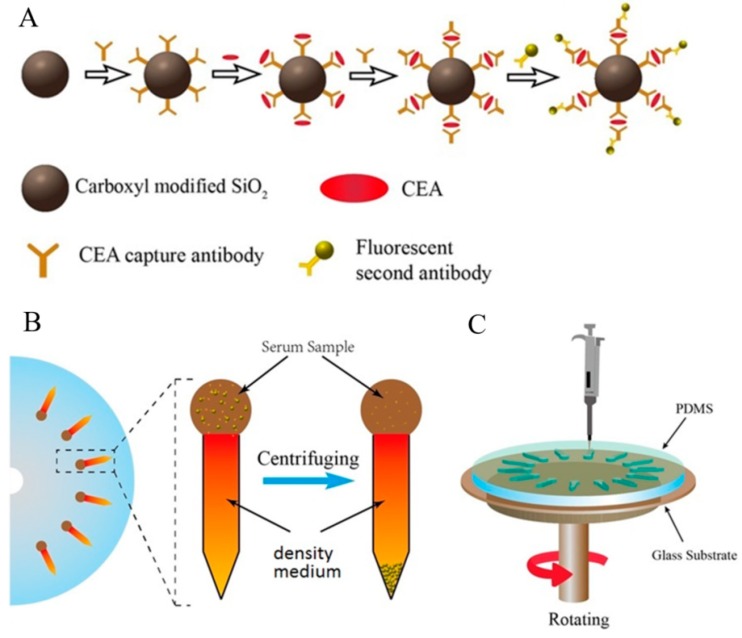
Design of the centrifugal microfluidic device. (**A**) schematic representation of the sandwiched immunocomplex formed by the binding of the target analyte; (**B**) schematic of the centrifugal microfluidic platform immunoassay, depicting the multiplexed analysis of the serum; (**C**) operating principle of the centrifugal microfluidic device.

A polydimethylsiloxane (PDMS, Sylgard 184, DowDuPont Inc., Midland, MI, USA) glass microfluidic device was designed, as shown in Figure 1A. The height, width, and length of the individual microchannels were 150 μm, 4.2 mm and 1.5 cm, respectively. The diameter of the inlet hole was 5 mm. The distance between the center of the chip and the inlet hole was 6.5 cm. With this geometry, the micro-channels were patterned in PDMS using replica molding. The mold was prepared by spin-coating a thin layer of negative photoresist (SU-8, MicroChem, Corp., Westborough, MA, USA) onto a single side of a polished silicon wafer, which was patterned using UV exposure. Next, the micro-channel layer was obtained by pouring PDMS with a 10:1 (*w*/*w*) base-to-crosslinker ratio onto the mold to a thickness of approximately 3 mm. After curing the elastomer for 2 h at 80 °C, the PDMS slab was peeled from the mold, and was then punched and hermetically bonded to a coverslip by plasma oxidation.

### 2.2. Medium Density Screening

Based on the sedimentation process, a theoretical calculation pertaining to the relationship between the density of a material and the centrifugal sedimentation has been proposed [16]. In the case of particle transport in fluids, as in a sedimentation processes, the particles are subject to a viscous force, called drag (*F*_d_). It is given by:

(1) $$F_{d}=C_{d}\frac{\rho_{\mathrm{fluid}}}{2}u^{2}A_{\mathrm{particle}},$$

where *ρ*_fluid_ and *u* are the density and velocity of the fluid relative to a particle, respectively, *A*_particle_ is the particle cross-sectional area, and *C*_d_ is the drag coefficient. In the laminar flow regime (Stoke’s drag), the drag coefficient is proportional to the fluid viscosity *μ* and inversely proportional to its velocity *u* relative to the particle, so that for a spherical particle with radius *r*, the drag force is

(2) $$F_{S}=6\pi \mu ru.$$

Based on this, materials with intermediate densities, but various viscosities were tested. The isolating effects of Percoll (Aladdin Inc., Los Angeles, CA, USA) at a concentration at 1.13 g/mL, 7% or 14% dextran (Aladdin Inc.), as well as 1% or 2% chitosan (Aladdin Inc.) were compared in this study. To start, 1.4 g of dextran and 0.01 g Poloxamer (127 F) were dissolved in 9.8 mL hot water, and then mixed with 0.2 mL 5% bovine serum albumin (BSA) solution and stored at 4 °C. Then, 2 g chitosan powder was dissolved in a 0.1 M hydrochloric acid solution for 24 h using a centrifugal mixer. Finally, the solution was used as the dense medium to be added to the centrifugal microfluidic channel.

### 2.3. Optimization of the Rotation Speed and Spin Time

After the channels of the microfluidic device were cleaned with Phosphate Buffered Saline (PBS), 10 μL of 1% BSA (Aladdin Inc.) were added, and the devices were stored in a refrigerator at 4 °C to block the protein binding sites on the PDMS. Prior to adding 5 μL of 2% chitosan into the microfluidic device, the channels were washed five times with PBS, and stored in a 4 °C refrigerator until just before use. Then, 30 μL carboxyl-modified silica microspheres (Mozhidong Ldt., Beijing, China) were added to a 500 μL centrifuge tube, diluted to 200 μL, and then packaged with 10 μL of the primary antibodies (18.1 g/mL, Abcam, London, UK). After incubation on a table concentrator at room temperature for 2 h, and being stored at 4 °C in a refrigerator overnight, the beads with antibodies became stabilized. Then, the bead–antibody complexes were washed three times with PBS (pH 7.4), and diluted to 200 μL. Then, 5 μL BSA at a concentration at 5% was added to the solution, which was incubated at room temperature on a table concentrator for 2 h to block the remaining protein binding sites. Then, 30 ng/mL CEA samples (Abcam), with labeled primary antibodies and fluorescence labeled secondary antibodies, were successively added into microchannels with syringes or pipettes. The microfluidic device was centrifuged at various angular velocities (1000, 1500, 2000, 2500, 3000, 3500 or 4000 rpm) or at 2500 rpm for various spin durations (60, 90, 120, 150, 180 or 240 s). Afterwards, fluorescence images were obtained using a fluorescence microscope (IX71, Olympus, Tokyo, Japan) that used a high-power mercury lamp as the fluorescence light source and an exposure time of 3.5 s. After the fluorescence images were obtained, the fluorescence intensity values, with the background subtracted, were read by ImageJ software (version 2.1, National Institutes of Health, Bethesda, MD, USA). Then, statistical analyses were performed based on the particular requirements.

### 2.4. Establishing a CEA Standard Curve

CEA antigen samples at various concentrations, with labeled primary antibodies and fluorescence-labeled secondary antibodies, were successively added and incubated. Finally, the microfluidic device was centrifuged for 150 s at 2500 rpm in a horizontal centrifuge. At the same time, fluorescence images were obtained using a fluorescence microscope with exposure time of 3.5 s. The fluorescence images were analyzed with ImageJ software to establish a standard curve between the concentration of CEA and the corresponding fluorescence intensity.

### 2.5. Detection of CEA in Human Serum

BSA blocked bead–antibody complexes, and labeled primary antibodies and fluorescence-labeled secondary antibodies were added to the centrifuge microfluidic device and incubated at room temperature for 2 h. Then, clinical serum samples, which were collected and provided by the Affiliated Hospital of Dalian Medical University from both healthy and cancer person, were added to the centrifuge chip, and spun at 2500 rpm for 2.5 min. After obtaining the fluorescence images using the fluorescence microscope and processing with the ImageJ software, the standard concentration curve were used to obtain the experimental CEA concentrations.

### 2.6. Statistical Analysis

The SPSS 18.0. (IBM, New York, NY, USA) was used for mean value and standard deviation calculation as well as significance testing.

## 3. Results

### 3.1. Design, Fabrication, and Verification of the Centrifugal Microfluidic Device

Figure 1 shows the centrifugal microfluidic platform for detecting CEA using a sedimentation-based immunoassay. The sample was mixed with a detection cocktail consisting of silica microbeads (1 μm diameter), which were coated with specific antibodies for the target of interest, in this case, CEA. The detection antibodies were labeled with a fluorescent tag, which binds to the capture beads in the presence of the corresponding antigen (Figure 1A). After the serum samples were mixed with the antibody-conjugated capture beads and fluorescent detection antibodies in solution, they were added to a preloaded dense medium. The beads were pushed to the bottom of the channel to form pellets by the centrifugal force. Eventually, the target analytes separated from the rest of the sample (Figure 1B). The entire process of CEA detection could be completed in one step, as shown in Figure 1C. The samples were added at the entrance of the centrifugal microfluidic platform, which was split into 14 radially arranged pencil-like microchannels. Then, the target analyte could be detected using a sedimentation-based immunoassay. This simple, one-step centrifugal microfluidic platform provides high analytical accuracy and repeatability, which cannot be achieved by processes that require multiple steps.

To validate the effectiveness of the chemiluminescence immunoassay used in this device, CEA standard samples were measured using a double-antibody sandwich ELISA with the conventional method and the centrifugal microfluidic device. The proposed method was shown to be equivalent to the conventional method based on the linearity of the response (see supplementary material in the supporting information).

### 3.2. Medium Screening and Structure Optimization of the Microfluidic Device

**Figure 2:**
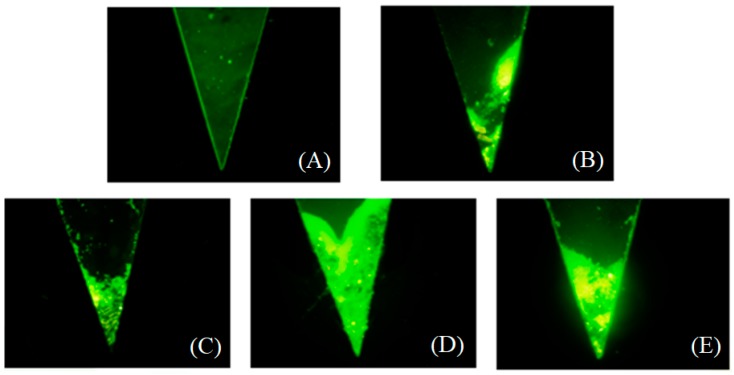

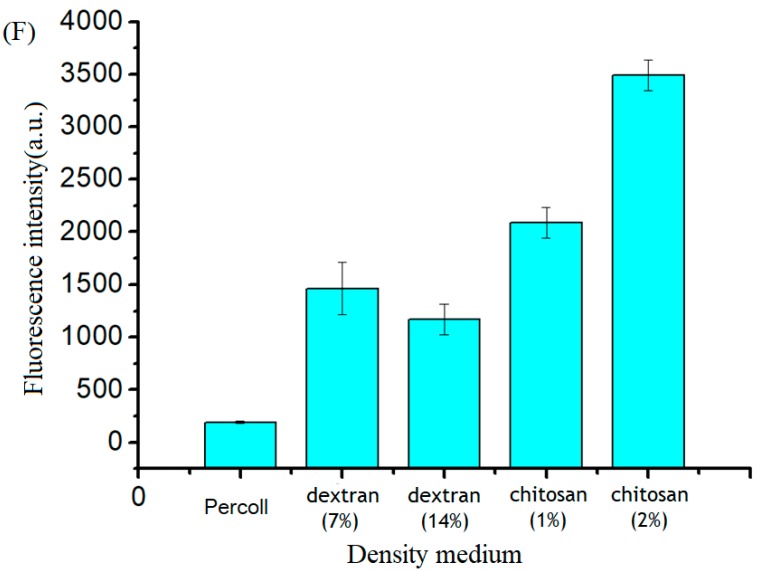
Effect of the dense medium on isolation efficiency in the centrifugal microfluidic platform. Separating effects of (**A**) Percoll, dextran ((**B**) 7% or (**C**) 14%), and chitosan ((**D**) 1% or (**E**) 2%) as dense media and (**F**) their histogram comparison.

Based on Equation (2), we screened various media to determine the optimal density and viscosity, as one of the critical aspects in this study. Percoll, dextran (7% or 14%), and chitosan (1% or 2%) were tested as dense media, as shown in Figure 2. It was suggested that the microbeads could not be separated in a Percoll solution at a concentration of 1.13 g/mL (Figure 2A). However, if the sample is whole blood with a density of 1.09 to 1.11 g/mL of red blood cells, Percoll can separate red blood cells from plasma. Although dextran can separate the microbeads from solution as a clinical plasma substitute, the separating effect is not as good as with the chitosan solution (Figure 2B,C). Because the concentration of the dense medium solution is 1%, the modified antigen antibody beads can become separated at the end of the microchannel. Furthermore, the rest of the solution will have been mixed with the medium because this is beyond the abilities of the separation process (Figure 2D,E). In addition, if the concentration of the dense medium is 2%, the modified antigen antibody beads can pass through the medium to reach the bottom of the channel. Therefore, a chitosan solution with a concentration of 2% was selected as the dense medium in the centrifugal microfluidic device to separate the microbeads modified by antigen antibodies in solution.

### 3.3. Effects of Rotation Speed and Spin Duration on the Detection of CEA

The effects of rotation speed and spin duration were also considered in this study. In the centrifugal microfluidic device with chitosan as the dense medium, the effects of various angular velocities on the isolation power of CEA standard samples (25 ng/mL) were investigated.

**Figure 3:**
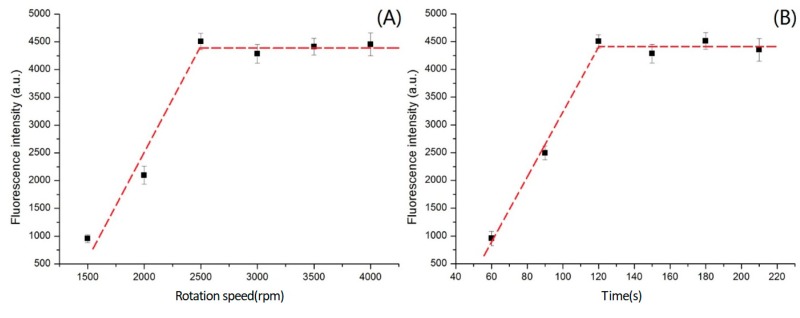
Effects of rotation speed and spin duration on carcinoembryonic antigen (CEA) isolation. (**A**) the effect of rotation speed on CEA isolation; (**B**) the effect of spin duration on CEA isolation.

In Figure 3A, the fluorescence intensity of aggregated microbeads increased as the rotation speed increased from 1000 to 2500 rpm. However, even if the rotation speed was set to greater than 2500 rpm, the fluorescence intensity of the aggregated microbeads did not increase, and instead plateaued at a constant value. This indicates that spinning at 2500 rpm caused all the microbeads in the bulk serum to migrate to the end of the microchannel. Thus, 2500 rpm was used as the rotation speed for CEA detection.

Similarly, the effects of various spin durations of the centrifugal microfluidic device using chitosan as the dense medium on the isolation of CEA in a standard sample (25 ng/mL) were investigated at 2500 rpm. As shown in Figure 3B, in the first two minutes, the microbeads were gradually separated to the end of the microchannel, and the fluorescence intensity increased over time. After two minutes, the microbeads in the sample were almost completely in the detection area, and the fluorescence intensity was constant thereafter. To have a margin of safety, 2.5 min was selected as the centrifugal spin duration.

### 3.4. CEA Detection in Human Serum Samples

**Figure 4:**
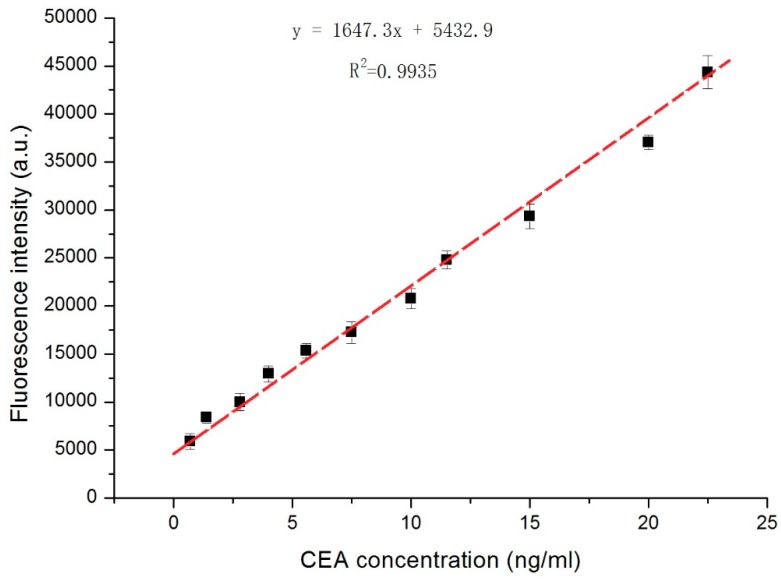
Relationship between fluorescence intensity of aggregated microbeads and CEA concentration in the serum.

CEA standard samples with various concentrations were measured using this device, and the relationship between CEA concentration (*x*) and the fluorescence intensity (*y*) was established with a standard curve (*y* = 1647.3*x* + 5432.9). It is suggested that the analytical sensitivity of the standard curve is 1673.4. Based on Figure 4, the repeatability at each concentration was good (*n* = 4), and the curve was linear within the CEA concentration range of 0.7–22.5 ng/mL (R^2^ = 0.993). Thus, these equations can be used as a standard curve for sample detection, including human serum samples.

**Figure 5:**
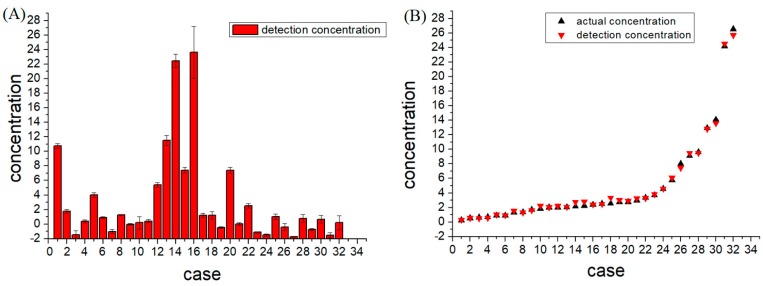
CEA detection in clinical human serum samples. (**A**) the repeatability of actual samples; (**B**) comparison of known and measured concentrations.

CEA in clinical human serum samples with known concentrations were measured using the centrifugal microfluidic device, and comparisons between the known and measured concentrations are shown in Table 1 and Figure 4. It can be seen from the table that the sample testing errors from 90% of the samples are less than 20%, and the average relative error was only 9.22%. This indicates that the detection results obtained by this device were reliable. In addition, as shown in Figure 5A, in these tests, excluding the poor repeatability of certain outlier samples, the repeatability of the remaining samples was good for CEA concentrations in the range of 0.5 ng/mL to 27 ng/mL. In addition, it was determined from Figure 5B that, when the carcinoembryonic antigen concentration was less than 2.0 ng/mL, the detection concentration was consistent with the known concentration, and when the concentration of the serum sample was higher than 10 ng/mL, there were some slight differences between the actual and measured concentrations.

The CEA detection results using the centrifugal microfluidic device and the hospital instruments were compared. As shown in Table 2, when excluding samples 7, 18 and 26, the *p*-value of samples were above 0.05. This suggests that there were no significant differences between the detection method of the centrifugal microfluidic device and the hospital’s method.

## 4. Discussion

A centrifugal microfluidic chip was constructed for detecting CEA in clinical serum samples. The device is based on a sandwiched immunoassay with biocompatible chitosan as the dense medium. It is driven by centrifugal force to implement rapid, high-throughput detection of serum CEA and other biomarkers. This centrifugal microfluidic chip does not require any washing steps, and can simplify the experimental procedure with increased accuracy and efficiency, compared with traditional immunoassays. Thus, this centrifugal microfluidic device can be expected to be used for CEA determination in hospitals.

The dense medium used in this microfluidic device was 2% chitosan to eliminate the need for the complicated washing steps of the traditional detection method. This device was able to separate the analyte from the solution in a one-step centrifugal process. Therefore, the samples could be detected directly and easily. In addition, the 14 individual pencil channels acted as parallel systems on the centrifugal microfluidic chip, with a relative standard deviation (RSD) value of just 4.95%.

The rotation speed and spin duration were optimized for the microfluidic device. It was determined that 2500 rpm for 150 s was able to completely move the analytes of the samples to the detection area. Thus, this microfluidic device can improve upon the current clinical detection methods.

Finally, the CEA detection results obtained from this centrifugal microfluidic device were compared and verified with clinical values. Thirty clinical serum samples were measured based on the standard curve established between the CEA concentration and fluorescence intensity. The detection error in 90% of the samples was less than 20% (Table 1), and the repeatability of the samples was good over the range of concentrations of 0.5 ng/mL to 27 ng/mL. Although a few samples showed low reproducibility, this might have been because the CEA was not uniformly distributed in the serum, and the volume of samples added to microfluidic device was microscale, thus making the contents to be measured in each sample unstable.

In conclusion, this study describes a centrifugal microfluidic device that was developed for detecting CEA. This method uses density gradient centrifugation and is free of washing steps, and is thus more accurate than traditional ELISA methods. It also can achieve high-throughput detection, with the potential to be used in central labs.

## Figures and Tables

**Table 1:** Results from 32 clinical serum samples.

No.	Known Value (ng/mL)	Measured Value (ng/mL), *n* = 3	Relative Error
1	12.94	12.82 ± 0.33	0.96%
2	3.69	3.82 ± 0.26	3.52%
3	0.71	0.58 ± 0.52	18.59%
4	2.4	2.43 ± 0.21	1.45%
5	5.76	6.10 ± 0.30	5.87%
6	2.75	2.94 ± 0.15	7%
7	0.91	1.03 ± 0.29	13.14%
8	2.55	3.31 ± 0.10	29.96%
9	2.01	2.04 ± 0.07	1.55%
10	2.02	2.23 ± 0.85	10.50%
11	2.52	2.47 ± 0.22	1.99%
12	8.01	7.46 ± 0.29	6.86%
13	14.07	13.59 ± 0.69	3.43%
14	24.16	24.54 ± 0.93	1.59%
15	9.12	9.48 ± 0.39	3.99%
16	26.54	25.69 ± 3.57	3.19%
17	3.35	3.29 ± 0.28	1.66%
18	2.98	3.27 ± 0.48	9.61%
19	1.34	1.57 ± 0.10	17.39%
20	9.63	9.48 ± 0.44	1.59%
21	2.04	2.08 ± 0.19	1.94%
22	4.58	4.60 ± 0.31	0.36%
23	0.91	0.93 ± 0.11	1.91%
24	0.53	0.55±0.15	4.67%
25	2.72	3.05 ± 0.37	12.31%
26	1.71	1.63 ± 0.46	4.48%
27	0.25	0.30 ± 0.08	21.68%
28	2.24	2.83 ± 0.57	26.23%
29	1.38	1.29 ± 0.18	6.55%
30	2.17	2.73 ± 0.51	25.8%
31	0.66	0.52 ± 0.36	21.22%
32	1.81	2.24 ± 0.97	24.02%

**Table 2:** *p*-values between paired samples.

Samples	*p*-Value	Samples	*p*-Value	Samples	*p*-Value
1	0.299	12	0.655	23	0.514
2	0.752	13	0.728	24	0.953
3	0.497	14	0.114	25	0.112
4	0.655	15	0.13	26	0.032
5	0.474	16	0.761	27	0.162
6	0.748	17	0.662	28	0.545
7	0.016	18	0.001	29	0.394
8	0.389	19	0.164	30	0.255
9	0.762	20	0.084	31	0.471
10	0.437	21	0.32	32	0.668
11	0.453	22	0.71	--	--

## Supplementary Materials

The following are available online at http://www.mdpi.com/2072-666X/9/9/470/s1, Figure S1: Chip channel parallelism measurement, Figure S2: Standard curve for determination of protein content, Figure S3: The comparation of fluorescence concentration curve between conventional ELISA method and centrifugal microfluidic device. (A) conventional ELISA method; (B) centrifugal microfluidic device.
