# Supplementary material for: Measurement of Carcinoembryonic Antigen in Clinical Serum Samples Using a Centrifugal Microfluidic Device
Source: Micromachines (Basel). 2018 Sep 17;9(9):470. doi: 10.3390/mi9090470 (PMC6187594; doi:10.3390/mi9090470)
Supplement: Supplementary file 1 [file micromachines-09-00470-s001.pdf]

Supplementary Materials: Measurement of Carcinoembryonic Antigen in Clinical Serum Samples using a Centrifugal Microfluidic Device

1. Parallel Validation of Chip Channel

Fourteen individual channels in one chip was added CEA with concentration at 30 ng/mL, after centrifugated at 3000 r/min for 5 mins, fluorescence intensity was detected as shown in Figure S1. It can be seen that the parallelism of channels were well, and the relative standard deviation (RSD) is only 4.95%. Thus, fourteen individual channels could be thought as same.

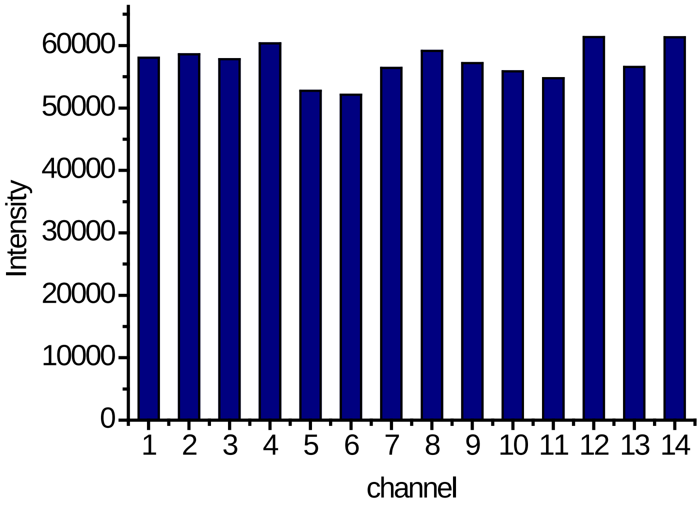

Figure S1. Chip channel parallelism measurement.

2. Protein Binding Rate Verification of Microbeads

The protein contents in standard protein samples and the solutions that before and after packaging microbead were detected by the kit and enzyme label instrument. The standard curve of protein was shown in Figure S2. The relationship between protein content and absorbance value was $y = 0.0498x + 0.021$, and $R^2 = 0.995 > 0.99$, and the results indicated that this curve could be used to determine the content of protein.

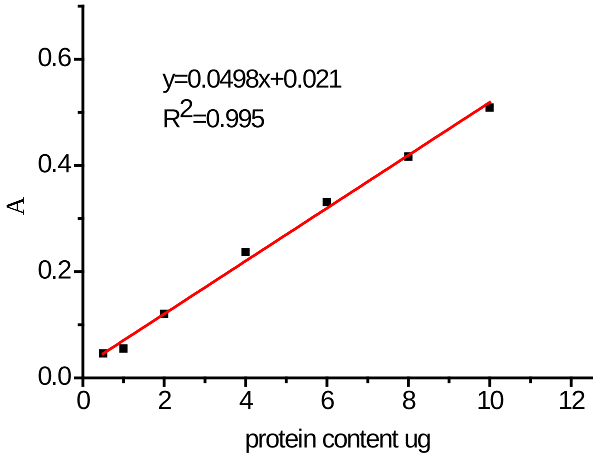

Figure S2. Standard curve for determination of protein content.

3. The Comparation of Fluorescence Concentration Curve between Conventional ELISA Method and Centrifugal Microfluidic Device

As shown in Figure S3, the concentration of CEA keep a linear relationship with fluorescence intensity between 0.7 to 11.25 ng/mL, and the values of R^2 are 0.986 and 0.982 respectively. It is observed that the intensity in centrifugal microfluidic device (Figure 3B) is equal to the conventional method in terms of linearity.

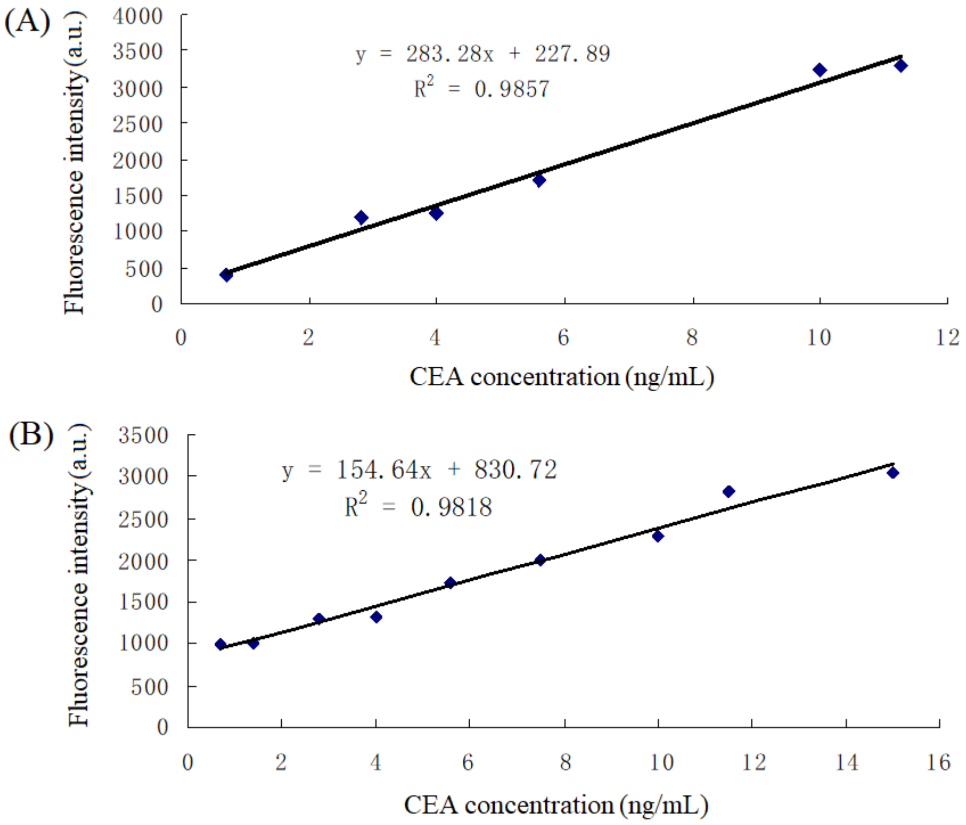

Figure S3. The comparation of fluorescence concentration curve between conventional ELISA method and centrifugal microfluidic device. (**A**) conventional ELISA method; (**B**) centrifugal microfluidic device.
